# Combining biophysical methods for the analysis of protein complex stoichiometry and affinity in *SEDPHAT*

**DOI:** 10.1107/S1399004714010372

**Published:** 2015-01-01

**Authors:** Huaying Zhao, Peter Schuck

**Affiliations:** a Dynamics of Macromolecular Assembly Section, Laboratory of Cellular Imaging and Macromolecular Biophysics, National Institute of Biomedical Imaging and Bioengineering, National Institutes of Health, Bethesda, MD 20892, USA

**Keywords:** GMMA, *SEDPHAT*

## Abstract

Reversible macromolecular interactions are ubiquitous in signal transduction pathways, often forming dynamic multi-protein complexes with three or more components. Multivalent binding and cooperativity in these complexes are often key motifs of their biological mechanisms. Traditional solution biophysical techniques for characterizing the binding and cooperativity are very limited in the number of states that can be resolved. A global multi-method analysis (GMMA) approach has recently been introduced that can leverage the strengths and the different observables of different techniques to improve the accuracy of the resulting binding parameters and to facilitate the study of multi-component systems and multi-site interactions. Here, GMMA is described in the software *SEDPHAT* for the analysis of data from isothermal titration calorimetry, surface plasmon resonance or other biosensing, analytical ultracentrifugation, fluorescence anisotropy and various other spectroscopic and thermodynamic techniques. The basic principles of these techniques are reviewed and recent advances in view of their particular strengths in the context of GMMA are described. Furthermore, a new feature in *SEDPHAT* is introduced for the simulation of multi-method data. In combination with specific statistical tools for GMMA in *SEDPHAT*, simulations can be a valuable step in the experimental design.

## Abstract 2

Global multi-method analysis for protein interactions (GMMA) can increase the precision and complexity of binding studies for the determination of the stoichiometry, affinity and cooperativity of multi-site interactions. The principles and recent developments of biophysical solution methods implemented for GMMA in the software *SEDPHAT* are reviewed, their complementarity in GMMA is described and a new GMMA simulation tool set in *SEDPHAT* is presented.

## 1. Introduction   

Protein interactions play essential roles in signaling pathways, transcriptional regulation and numerous other biological processes (Gavin *et al.*, 2002 ▶; Matthews, 2012 ▶). They mediate the formation of reversible complexes of proteins with other macromolecules such as proteins, nucleic acids and/or small molecules to generate structures and biological responses. Protein interaction networks determined from proteomic experiments have been shown to involve thousands of proteins with known or unknown functions, most of them contributing to large complexes containing many subunits (Gavin *et al.*, 2002 ▶). In order to understand the biological processes regulated by these protein interactions, we need to investigate the dynamics of these interactions and to obtain detailed information on the composition (stoichiometry) of the complexes, their physicochemical driving force (binding free energy) and their information transfer (binding cooperativity). These thermodynamic parameters represent the basic functional characteristics of an interacting system, and are complementary to structural elucidation using crystallo­graphy. In particular, the binding free energy and its enthalpic and entropic components directly relate to the nature of the binding interface and have been widely used in the pharmaceutical industry as markers for drug development.

In the past decades, with the assistance of various biophysical methods, such as analytical ultracentrifugation (AUC), isothermal titration calorimetry (ITC), surface plasmon resonance biosensing (SPR), nuclear magnetic resonance (NMR) and fluorescence spectroscopy, the stoichiometry and affinity of many protein interactions have been measured. However, using any of these methods, the study of complex multi-component or multi-site systems can be highly challenging. On the other hand, such interactions are ubiquitous and of great interest and, in particular, the cooperativity between the different binding interfaces is a key feature of protein function.

The concept of global analysis of protein interaction data from multiple experiments was described soon after the introduction of computer-aided data analysis, and has been demonstrated to help to increase the information content of the data (Knutson *et al.*, 1983 ▶; Beechem, 1992 ▶), similar to hybrid methods in structure determination (Robinson *et al.*, 2007 ▶). To this end, in the last decade we have developed the software *SEDPHAT* as a computational engine for globally analyzing multiple data from different biophysical methods. It has a flexible and user-friendly graphical interface that does not require any system-specific or data-dependent programming. *SEDPHAT* was first established for the global analysis of multiple data sets from a single technique. For ITC, we have demonstrated how this can substantially improve the level of detail and reveal the cooperativity parameters in ternary multi-protein complexes (Houtman *et al.*, 2007 ▶); in sedimentation equilibrium analytical ultracentrifugation (SE) *SEDPHAT* has been routinely used in global analysis to overcome the limitation of ill-conditioned SE data and obtain better determination of thermodynamic parameters (Vistica *et al.*, 2004 ▶; Ghirlando, 2011 ▶; Zhao, Brautigam *et al.*, 2013 ▶); and multiple approaches for global analysis in sedimentation velocity analytical ultracentrifugation (SV) include direct boundary modeling with Lamm equation solutions for determining equilibrium and kinetic binding constants, as well as hydrodynamic shape parameters (Schuck, 2003 ▶; Dam *et al.*, 2005 ▶; Brautigam, 2011 ▶), multi-signal analysis (MSSV) for determining the number and composition of co-existing complexes (Balbo *et al.*, 2005 ▶; Padrick & Brautigam, 2011 ▶; Brautigam *et al.*, 2013 ▶) and global density-contrast analysis for determining macromolecular partial specific volume (Brown *et al.*, 2011 ▶). Finally, applications of global analysis of combined SE and SV data in *SEDPHAT* have demonstrated the utility of a more generalized sedimentation analysis (Canzio *et al.*, 2013 ▶; May *et al.*, 2014 ▶).

For the global analysis of multiple data sets from different biophysical methods (GMMA), we have recently introduced a few specific statistical functions in *SEDPHAT* to address the combination of data sets of dissimilar size and information content (Zhao & Schuck, 2012 ▶). As demonstrated with a model system for a two-site binding process (Zhao & Schuck, 2012 ▶), GMMA can significantly improve the precision and resolution of thermodynamic analyses of multi-site systems. A recent application to a three-site system can be found in Gustchina *et al.* (2013 ▶), which highlights the advantage of GMMA over single-technique data that can be hard to interpret alone. In the present paper, we provide a brief overview of the basic principles and the most recent developments of select biophysical methods for the study of protein interactions, with special emphasis on their complementarity in the context of GMMA. We then describe a new function implemented in *SEDPHAT* to simulate multi-method data in order to facilitate the experiment planning and to solidify data interpretation for complex interacting systems. With the improvements in sensitivity and resolution of the biophysical methods, and the new computational tools in *SEDPHAT*, the GMMA approach allows us to gain new perspectives for studying complex interactions and to further propel understanding of biological functions.

## 2. Recent developments in SV, ITC and SPR for protein binding studies   

### 2.1. Sedimentation-velocity analytical ultracentrifugation (SV)   

Analytical ultracentrifugation (AUC) is a classical and first-principle-based technique for characterizing macromolecules and nanoparticles in solution, and has a long history of applications to biological macromolecules and their reversible interactions (Schachman, 1959 ▶). The basic objective of the analytical ultracentrifuge is to monitor and interpret the evolution of the macromolecular concentration profiles after the application of a centrifugal field. For a basic introductory review and practical protocols, see, for example, Lebowitz *et al.* (2002 ▶) and Zhao, Brautigam *et al.* (2013 ▶). Sedimentation velocity (SV) and sedimentation equilibrium (SE) are the two standard experimental designs in AUC, with SV focusing on the sedimentation process while SE examines the final equilibrium distribution. For protein studies, the combination of these two methods can provide powerful information of protein size, size distribution and purity, hydrodynamic shape and affinity for binding other macromolecules (Zhao, Brautigam *et al.*, 2013 ▶). AUC can be used to study a wide range of particle sizes from 100 to 10^8^ g mol^−1^. Currently, three types of optical systems are available for AUC: the conventional absorbance spectrophotometer, the Rayleigh interferometer and the recently commercially introduced fluorescence-detection system (FDS). With different optical detection systems and different experimental approaches, AUC offers a remarkably broad dynamic range for investigating protein interactions with equilibrium dissociation constants (*K*_d_) from picomolar to millimolar (Chaudhry *et al.*, 2009 ▶; Rowe, 2011 ▶; Zhao *et al.*, 2014 ▶). The application of AUC to protein binding studies allows determination of the stoichiometry and the affinity of protein complex formation, including both self-association and hetero-association.

SV, in particular, is highly advantageous in the study of protein interactions, mainly because the strong size-dependent sedimentation process leads to high hydrodynamic resolution, which is usually far superior to diffusion-based methods such as dynamic light scattering or size-exclusion chromatography. Furthermore, in the standard experimental design of SV, despite their higher sedimentation velocity the protein complexes will remain in a bath of their slower sedimenting constituent components, such that dissociating complexes can re-associate during the sedimentation process in a way that reflects their equilibrium and kinetic properties. In recent years, it has undergone substantial improvements in instrumentation, theory and computational data analysis, which have benefited numerous protein studies for deciphering the composition of protein complexes, binding mechanisms and specificity (Schuck, 2013 ▶). Here, we focus on some of the most recent developments in SV relevant to protein interaction studies.

#### 2.1.1. Diffusion-deconvoluted and spectrally deconvoluted sedimentation coefficient distributions *c*(*s*) and *c*_*k*_(*s*)   

One of the most valuable aspects of SV is the great hydrodynamic resolution and sensitivity that can be applied to determine the number, the size and the hydrodynamic shape of co-existing protein complexes. A critical advance in SV was made in the 1990s with the ability for routine efficient numerical solution of the master equation of sedimentation and diffusion fluxes in the sector-shaped solution column: the Lamm equation (Lamm, 1929 ▶; Schuck, 1998 ▶; Brown & Schuck, 2007 ▶). This provides a model for the temporal and spatial evolution of the concentration of a single non-interacting particle χ_1_(*r*, *t*) and opened the door to the direct modeling of the observed sedimentation boundaries *a*(*r*, *t*).

The extension of this to the description of a coupled sedimentation of a kinetically interacting system has been developed (Schuck, 1998 ▶, 2003 ▶; Stafford & Sherwood, 2004 ▶; Dam *et al.*, 2005 ▶). In principle, it can provide binding constants, the sedimentation coefficients (*s*-values) of all species and – under highly favorable conditions of reaction kinetics on the same timescale as the sedimentation experiment, *i.e.* with complex lifetimes of approximately 1 h – estimates for the kinetic rate constants for chemical interconversion. While conceptually very powerful, it has the important drawback of requiring highly pure sample and prior knowledge of (or a hypothesis on) the complexes formed (Brautigam, 2011 ▶; Zhao *et al.*, 2011 ▶; Zhao, Brautigam *et al.*, 2013 ▶).

A more widely applicable approach, which is usually the first step in modern data analysis, is the combination of single-species Lamm equation solutions χ_1_(*s*, *r*, *t*) into sedimentation-coefficient distributions *c*(*s*). It is defined by the integral
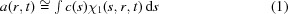
that is fitted directly to the set of observed boundary profiles. It is important to take precautions against overfitting through the use of maximum entropy or Tikhonov regularization (Schuck, 2000 ▶), which may be tailored to specific prior knowledge (Brown *et al.*, 2007 ▶). Similar to the deconvolution of point-spread functions in optical imaging, *c*(*s*) results in diffusion-deconvoluted sedimentation-coefficient distributions.

In most common cases, the biological sample solution is composed of an ensemble of molecules with various sizes. Such polydispersity is challenging in many biophysical methods. However, the *c*(*s*) analysis can detect and account for this: the high hydrodynamic resolution and sensitivity to trace components allow unrelated sedimenting species to be excluded from further analysis and therefore prevent inconsistencies in GMMA arising from different sensitivity to impurities. For sufficiently long-lived complexes, *c*(*s*) can resolve the number of co-existing complexes and determine the molecular weight and *s*-value (*i.e.* a molecular shape function). For short-lived complexes, *c*(*s*) provides a platform for efficient further thermodynamic analysis (see below).

The stoichiometry of multi-component complexes can be directly resolved from sedimentation velocity data with multiple signals, if it is possible to exploit different spectral signatures of different components. For example, the spectral difference could be owing to different aromatic amino-acid contents causing differences in the extinction coefficients at 280 or 250 nm, or in combination with refractive-index signals, or using chromophoric labels in the visible-light region. We can take advantage of such differences in the multi-signal sedimentation coefficient distribution *c_k_*(*s*),
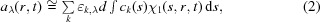
(where ∊_*k*,λ_ represents the extinction coefficient of component *k* at wavelength λ and *d* is the optical path length), which simultaneously fits the data *a*_λ_(*r*, *t*) acquired at the different signals and spectrally convolutes component contributions (Balbo *et al.*, 2005 ▶). The integration of *c_k_*(*s*) reports the concentration of components co-sedimenting in a certain peak. Details for the application of this multi-signal SV (MSSV) approach and requirements for spectral resolution can be found in Padrick & Brautigam (2011 ▶), Brautigam *et al.* (2013 ▶) and Zhao, Brautigam *et al.* (2013 ▶).

**Figure 1:**
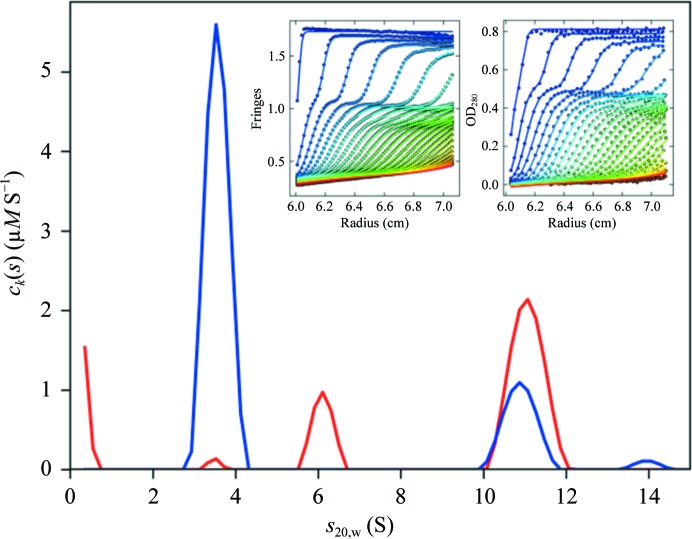
Example of the MSSV analysis of MHC class I (blue) binding to HHV-7 U21 (red) forming mixed 4:2 complexes (May *et al.*, 2014 ▶). The insets show raw boundary profiles from the Rayleigh interference optical system (left) and absorbance system at 280 nm (right). No chromophoric label was required for the spectral decomposition owing to the difference in content of aromatic amino acids in the two proteins.

The importance of MSSV in the context of protein interaction analysis is its capability to directly reveal the size and the composition of protein complexes, which can be invaluable prior information when modeling binding isotherms observed with other methods (Fig. 1 ▶), especially for multi-component systems. This unique advantage has been demonstrated in various multi-component protein systems (Houtman *et al.*, 2006 ▶; Brautigam *et al.*, 2009 ▶; Barda-Saad *et al.*, 2010 ▶; Padrick *et al.*, 2011 ▶; May *et al.*, 2014 ▶).

#### 2.1.2. Isotherms of signal-weighted average sedimentation coefficients and effective particle theory   

The *c*(*s*) distribution can be integrated to determine signal-weighted average sedimentation coefficients, *s*_w_, which are directly related to the average overall transport of the interacting system (Rivas *et al.*, 1999 ▶; Schuck, 2003 ▶). When acquired as a function of solution composition, it provides a binding isotherm from which equilibrium constants can be determined, as well as the hydrodynamic shape of the individual species (Zhao, Brautigam *et al.*, 2013 ▶). Such isotherms can be easily loaded into *SEDPHAT* and fitted using nonlinear regression analysis with the appropriate binding model.

Furthermore, interacting systems at nonstoichiometric concentrations in SV produce characteristic boundary patterns that depend on the lifetime of the complexes relative to the duration of the SV experiment. For long-lived complexes, the boundary pattern directly reflects the population of different species, which often can be hydrodynamically resolved. For short-lived complexes, the sedimentation is coupled to the chemical interconversion, leading to the formation of effective particles that consist of free and complex species in proportions that ensure that the time-average sedimentation velocities of all macrocmolecules are equal (Schuck, 2010*a* ▶,*b* ▶). As we have shown recently, these characteristic boundary amplitudes, and their *s*-values, can also be assembled into isotherms that reflect equilibrium constants as well as species sizes and shapes, and in combination with the *s*_w_ isotherms increase the information content and precision of the binding analysis (Zhao *et al.*, 2011 ▶).

#### 2.1.3. Fluorescence-detected sedimentation velocity (FDS-SV)   

**Figure 2:**
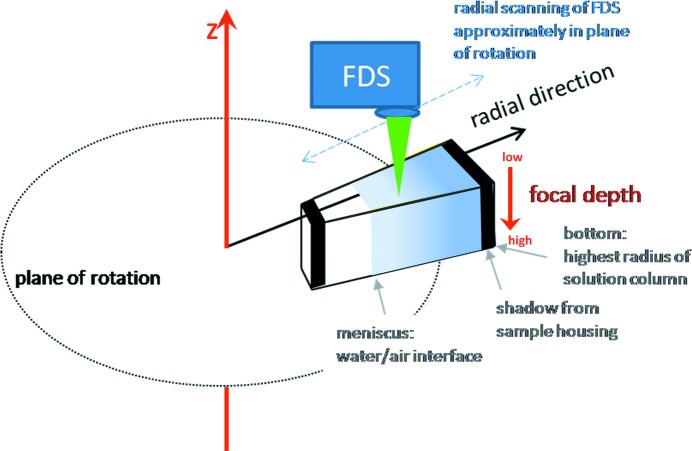
Schematic representation of the scanning setup of the FDS optics for AUC (MacGregor *et al.*, 2004 ▶). This figure was taken from Zhao, Casillas *et al.* (2013 ▶).

While traditionally AUC is constrained to the analysis of moderate to weak interactions with *K*_d_ values in the micromolar to millimolar range, based on the required optical detection by absorbance or interferometry, fluorescence detection can extend the dynamic range to nanomolar or even picomolar concentrations (Crepeau *et al.*, 1976 ▶; Schmidt *et al.*, 1990 ▶; Schmidt & Riesner, 1992 ▶; MacGregor *et al.*, 2004 ▶). The fluorescence-detection system (FDS) resembles a moveable confocal microscope featuring a focal point that scans radially through the solution column (Fig. 2 ▶). A design by Laue and coworkers (MacGregor *et al.*, 2004 ▶) has recently become commercially available from AVIV Biomedical Inc. (Lakewood, New Jersey, USA).

**Figure 3:**
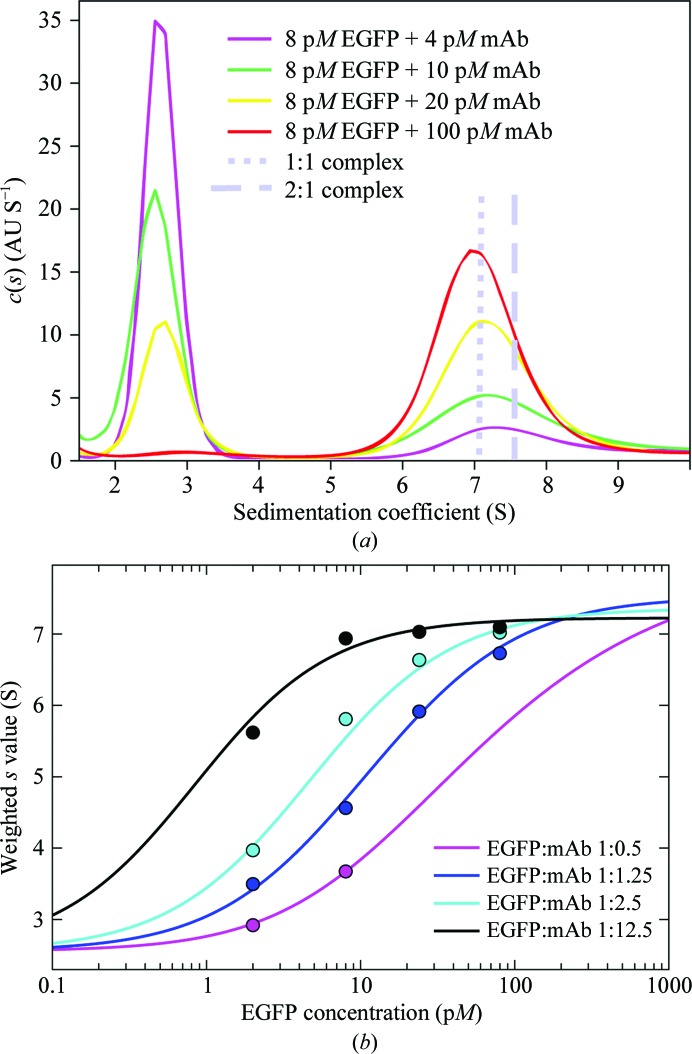
High-affinity interaction of an EGFP and GFP monoclonal antibody (Zhao *et al.*, 2014 ▶): (*a*) *c*(*s*) distributions from FDS-SV data; (*b*) *s*_w_ isotherms (symbols) and best-fit model (lines) at different macromolecular concentrations, leading to a microscopic binding constant of 20 p*M*.

Currently, excitation is restricted to 488 nm and emission is acquired in a wavelength band from 505 to 565 nm. This provides optimal sensitivity for GFP and FITC-related dyes, but owing to the exquisite sensitivity of the commercial system detection of some red fluorophores is also possible (unpublished observation). Initially, the majority of applications of FDS-SV were of a qualitative rather than a quantitative nature (Kroe & Laue, 2009 ▶; Kingsbury & Laue, 2011 ▶), but the analysis of FDS-SV data has undergone rapid development (Bailey *et al.*, 2009 ▶; Lyons *et al.*, 2013 ▶; Zhao, Casillas *et al.*, 2013 ▶; Zhao, Lomash *et al.*, 2013 ▶; Zhao *et al.*, 2014 ▶). In particular, we have recently shown that, after accounting for some characteristic data features including spatial and temporal changes in the signal magnification, highly quantitative fits can be achieved with signal-to-noise ratios rivaling that of the best traditional optical system (Zhao, Casillas *et al.*, 2013 ▶). Furthermore, when fully exploiting the very large statistics of data points that can be acquired in FDS-SV, and when using an inert carrier protein to block surface adsorption (after appropriate controls), it is possible to measure sedimentation coefficients of protein complexes at low picomolar EGFP concentrations, which opens this method up to the study of ultrahigh affinities with subnanomolar equilibrium constants (Zhao *et al.*, 2014 ▶; Fig. 3 ▶). In the context of GMMA, this brings the sensitivity of SV-AUC onto a par with (and even exceeding) that of SPR and ITC.

#### 2.1.4. Detergent-solubilized membrane proteins and nanodiscs   

It is important that the AUC methods described above are equally as suitable for soluble proteins as for membrane proteins. While the study of detergent-solubilized membrane proteins was pioneered by Reynolds and Tanford based on SE (Reynolds & Tanford, 1976 ▶), modern SV analysis capabilities and new solubilization strategies have stimulated significant progress in recent years (Ebel, 2011 ▶). In either of these approaches, additional steps in experimental planning and data analysis are required to account for the contributions of detergent to the sedimenting macromolecule (Ebel, 2011 ▶). Furthermore, AUC is completely compatible with membrane proteins reconstituted into nanodisc systems (Inagaki *et al.*, 2012 ▶; Monterroso *et al.*, 2013 ▶). These developments advance the membrane protein studies and open the door to future GMMA applications by the combination of SV with other biophysical methods.

### 2.2. Isothermal titration microcalorimetry (ITC)   

ITC has served as a key technique for quantifying binding affinity and stoichiometry of protein interactions. It is based on the direct measurement of the reaction heat during a titration series of injections of a reactant into a thermally isolated vessel containing reaction partner(s). From the shape of the resulting isotherm of observed heats, the change of enthalpy, Δ*H*, the equilibrium constant can be determined and, traditionally, an ‘*n*’ value reflecting the reaction stoichiometry and active concentration errors. Numerous ITC applications have been accomplished in various systems with a range of *K*_d_ between 1 n*M* and 100 µ*M* (Wiseman *et al.*, 1989 ▶). In order to expand the accessible affinity range, various approaches have been developed using displacement or competition strategies (Sigurskjold, 2000 ▶; Velazquez Campoy & Freire, 2006 ▶; Krainer *et al.*, 2012 ▶). The required three-component binding models have been implemented as part of the ITC model set in *SEDPHAT*. In the context of multi-site interactions and GMMA, one unique feature of ITC is its suitability for detecting cooperativity in binding, because we observe both changes in enthalpy (ΔΔ*H*) and free energy (ΔΔ*G*) (Bains & Freire, 1991 ▶; Houtman *et al.*, 2007 ▶).

#### 2.2.1. Global modeling of titration isotherms   

In the last decade, it has become abundantly clear that ITC is significantly more powerful when multiple titrations are analyzed globally (Henzl *et al.*, 2003 ▶; Armstrong & Baker, 2007 ▶; Houtman *et al.*, 2007 ▶; Freiburger *et al.*, 2009 ▶; Herman & Lee, 2009 ▶; Coussens *et al.*, 2012 ▶). This is true for simple repetitions as well as for the combination of separately suboptimal experiments into a well defined global analysis. This is even more critical when studying systems with more than two components and more than two sites, such as multi-protein complexes, and whenever multiple titrations can sample the *n*-dimensional binding isotherm of *n*-component systems in orthogonal ways (Houtman *et al.*, 2007 ▶).

*SEDPHAT* is naturally capable of accommodating such global analyses in a flexible manner with a menu-driven or a drag-and-drop interface. The growing list of global ITC analysis models includes various two-component and three-component protein interactions, with macroscopic or microscopic descriptions to exploit known symmetries and competition models, as well as salt-dependent, temperature-dependent and protonation-linked binding models.

A prerequisite for the global analysis of ITC data is to abandon the concept of a nonphysical (and usually non-integral) ‘*n*’ value as a catch-all parameter for concentration errors, incompetent protein fractions and reaction stoichiometry. In the global context, as implemented in *SEDPHAT*, the reaction stoichiometry is fixed in the reaction model but separate parameters allow for concentration errors and/or incompetent fractions of material. The concentration errors can have lower and upper bounds, and may be linked across different experiments (‘linked local parameters’), where justified by experimental design and sample preparation.

#### 2.2.2. Integration with peak-shape analysis and error estimates   

Over the last decades, significant improvements in the instrumentation of ITC have allowed smaller sample volumes and more sensitive detection. However, baseline assignment for the power traces followed by peak integration to determine the heat of each injection is a nontrivial first step in the ITC analysis, and is often one of the limiting factors for data interpretation. This has a particularly pronounced impact on the binding processes associated with smaller heats, and is exacerbated for smaller volume instruments. Since the manufacturer-provided automated integration algorithm cannot adapt well to the stochastic nature of the baseline drift and its adventitious jumps, it has previously been regarded the state-of-the-art strategy to manually adjust the baseline assignment (Velazquez Campoy & Freire, 2006 ▶). Clearly this is unsatisfactory, since it is subjective and potentially associated with bias. (This may not always be obvious since the assigned baseline is usually subtracted out in the final thermogram plots.)

**Figure 4:**
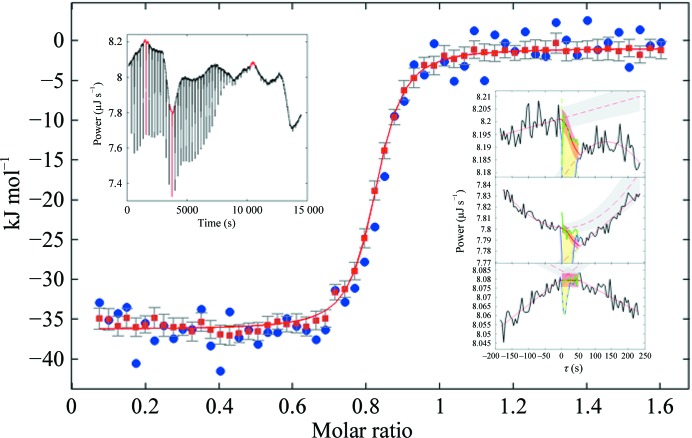
Titration isotherm from data after automated thermogram processing using *MicroCal Origin* (blue circles) or *NITPIC* (red squares and error bars) and fit (red line) to a 1:1 binding model of a *NITPIC* isotherm (from Keller *et al.*, 2012 ▶). The left inset shows the thermogram. The right insets illustrate the principle of baseline interpolation for three different injections as highlighted in red in the thermogram plot.

In order to develop an objective approach for ITC peak integration, we introduced a new method implemented in the *New Integrator of Thermograms Produced by Isothermal Calorimetry* (*NITPIC*; Keller *et al.*, 2012 ▶). Conceptually, this approach is based on the recognition that the peak shapes from all injections are similar (although not identical) to each other, such that regularization by truncated singular value decomposition can be used to distinguish baseline from injection heats (Keller *et al.*, 2012 ▶). This approach is particularly robust with regard to adventitious baseline jumps. The algorithm is self-adjusting to the noise in the baseline and usually runs fully automatically. Some algorithmic adjustments are possible, for example, in rare cases, to set overall threshold levels or to adapt criteria to nonstandard isotherms, but by design no adjustment to individual injections is possible (nor is it necessary). As illustrated in Fig. 4 ▶, it greatly outperforms the standard integration routines currently provided by the instrument manufacturers.

In addition to the isotherm of reaction heats, *NITPIC* determines error bars associated with the calculated heat of each injection on the basis of the baseline noise in the power trace surrounding the injection. This is important as it leads to proper statistical weighting in the isotherm analysis. For example, this allows realistic analysis of the statistical accuracy of binding parameters where very few data points are in the transition to saturation. This region of the data governs the estimate of the binding constant and will be sparsely sampled for high-affinity systems (high ‘*c*’-value conditions), and may therefore be sensitive to variable uncertainties of individual injection heats. Also, proper error estimates are of particular importance for the global ITC analysis when data with different loading concentrations in the reaction vessel (different ‘*c*’ values) are analyzed jointly, which produce very dissimilar statistical errors.

### 2.3. Surface plasmon resonance surface binding (SPR)   

Optical biosensing has become a popular technique for studying protein interactions with the introduction of a commercial SPR biosensor (Löfås *et al.*, 1991 ▶). In the most commonly used flow design, a protein is immobilized on a polymeric support at the sensor surface and its binding partner (or reaction mixture) is flowed across the sensor surface at various concentrations while monitoring the accumulation of surface-bound material optically *via* the solution refractive-index changes in the evanescent field of light in total internal reflection (Schuck, 1997 ▶). Analogous to the potential impact of the covalent attachment of extrinsic fluorophores to proteins in fluorescence techniques (including FDS-SV; Zhao, Lomash *et al.*, 2013 ▶), covalent attachment of the protein to the surface and the proximity of the surface can lead to alterations in its binding properties. Whether or not the surface binding parameters are identical to those in solution is strongly dependent on the particular proteins under study, and examples of both are common (Schuck *et al.*, 1998 ▶).

**Figure 5:**
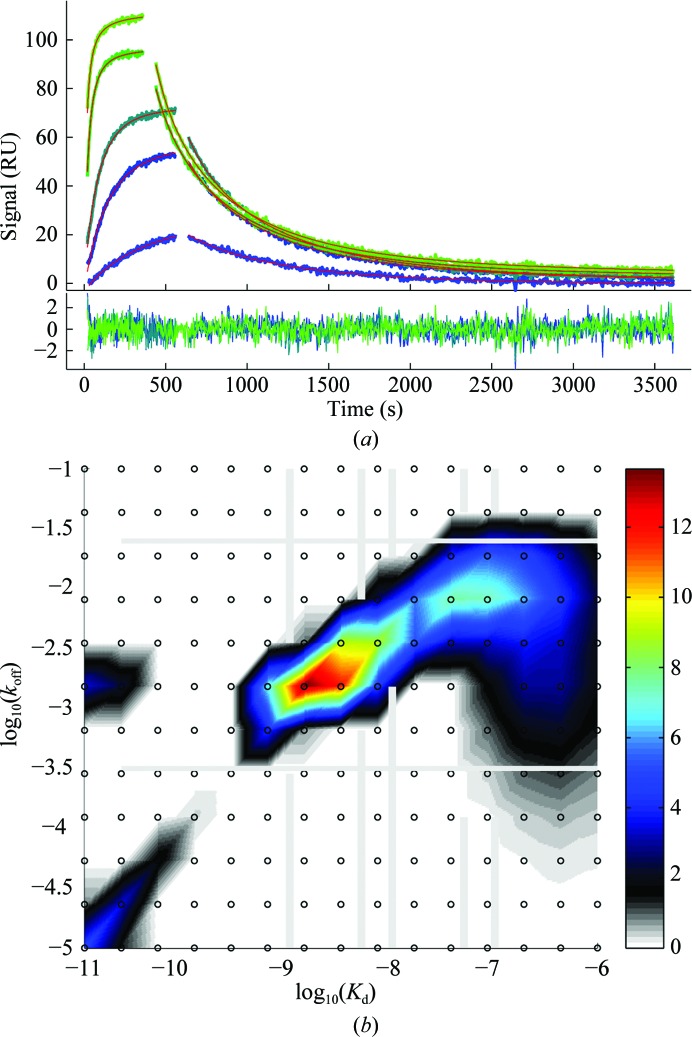
Example of SRP data analysis with a surface site distribution in *EVILFIT* (Zhao *et al.*, 2012 ▶). (*a*) Binding data (blue to green) and best fit (red lines) from the distribution model of an experiment of β_2_-microglobulin (1.0, 5.0, 10, 50 and 100 n*M*) binding to a CM5 sensor chip with anti-β_2_-microglobulin-biotin immobilized; (*b*) best-fit affinity and kinetic rate constant distribution from global fit of all data in (*a*).

The SPR biosensing data could directly offer kinetic information on the binding; however, very frequently the kinetics of surface binding significantly deviate from the expected pseudo-first-order theoretical model. This can be attributed to mass-transport limitation and/or variations of the physicochemical microenvironment of the sensor surface causing heterogeneity of the surface binding sites (Schuck & Zhao, 2010 ▶; Zhao *et al.*, 2012 ▶). To account for these issues from surface binding, a transformation of surface-binding progress data into a space of two-dimensional rate-constant distributions has been developed and implemented in the free software *EVILFIT* (Svitel *et al.*, 2007 ▶; Schuck & Zhao, 2010 ▶). *EVILFIT* typically leads to excellent fits of the raw data and the most populated or putatively native interactions represented in the major peaks to be focused on (Fig. 5 ▶). Tikhonov and Bayesian regularization allow the user to probe the information content of the SPR data.

In the context of GMMA these surface-induced artifacts of impaired binding sites pose a significant difficulty. Therefore, although *SEDPHAT* does allow the incorporation of steady-state surface-binding isotherms, in order to probe true solution interactions it is advantageous to conduct solution competition experiments, conceptually just like the competition experiments between labeled and unlabeled macromolecules in fluorescence approaches. We can exploit the SPR surface solely as a measure of the free binding partner in solution, which can be empirically calibrated through an initial series of surface-binding experiments. This is followed by experiments in which reaction mixtures are injected, which, dependent on the solution interaction, deplete the concentration of free surface-binding partner. Such a surface-competition isotherm is the preferred approach for SPR analysis in *SEDPHAT* in the context of global analysis. Indeed, global analysis of direct and competition isotherms can be performed to reveal differences between surface and solution binding (Schuck *et al.*, 1998 ▶).

A strength of the SPR analysis is its application to high-affinity interactions with *K*_d_ in the nanomolar range. However, a disadvantage that is important to keep in mind is that multivalent surface binding cannot be studied reliably, and the method is limited in the presence of self-associations. Furthermore, control experiments are essential for all solution components other than the specific binding partner to ensure that they do not bind to the surface.

## 3. Global multi-method analysis (GMMA) in *SEDPHAT*   

### 3.1. Basic principle of GMMA   

The goal of GMMA is to exploit synergies of the different biophysical techniques. The first step in the analysis of an interacting system is the definition of the thermodynamic states, *i.e.* the number and stoichiometry of complexes. In our experience, SV and MSSV often provide unique opportunities as they deliberately depart from the premise of a binding model upfront. If the binding scheme cannot be identified directly, it may be assessed later by evaluating the implications of different structurally motivated hypothetical models on the quality of fit of the binding data, with GMMA offering the most stringent criteria.

**Figure 6:**
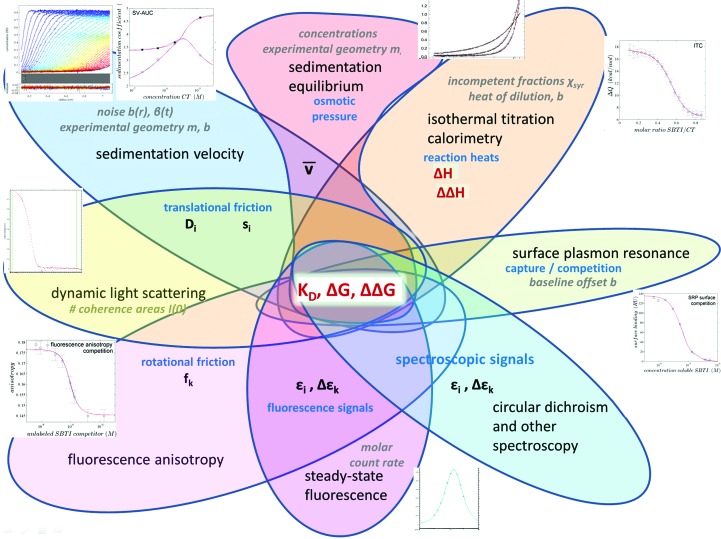
Venn diagram of different biophysical methods that can contribute to GMMA in *SEDPHAT*. Hierarchy of parameters: macromolecular binding parameters that are central TO the model for each data type, such as *K*_d_, Δ*G* and ΔΔ*G*, are indicated in bold red; macromolecular parameters that serve as observables in each technique, such as mass in AUC and SPR, translational frictional coefficient in SV and DLS, spectroscopic changes upon binding in fluorescence or other spectroscopy and rotational diffusion coefficient in fluorescence anisotropy, are indicated in bold black. In ITC, the enthalpic changes (Δ*H* and ΔΔ*H*) as binding parameters are directly probed calorimetrically. Finally, in most methods there are technical and ‘nuisance’ parameters that are usually unrelated to the molecules under study, such as baseline offsets and sample dimensions (meniscus and/or bottom in SV), but also incompetent fractions, as indicated in grey. Some of these local parameters may be constrained to be the same in a subset of experiments, such as concentration errors. A given model is projected into each of the data spaces, optimizing the local and nuisance parameters, compared with the experimental data, and a global measure for the goodness of fit is calculated, which is then optimized by nonlinear regression.

**Figure 7:**
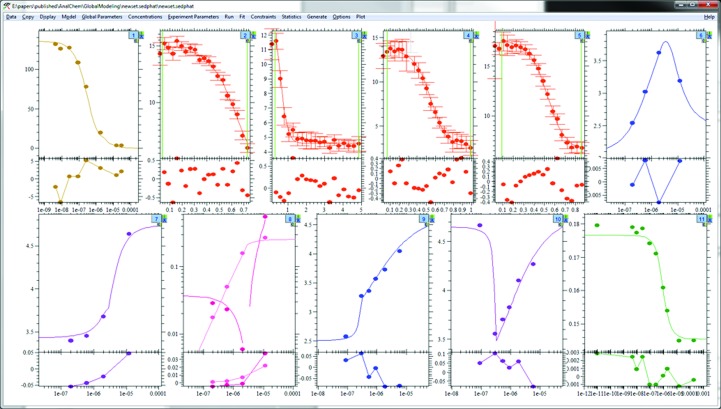
Screenshot of GMMA fit of 11 data sets from four biophysical methods of binding between α-chymotrypsin and soybean trypsin inhibitor from Zhao & Schuck (2012 ▶). Top row, left to right: SPR competition experiment, four ITC titrations, *s*_w_ isotherm from a dilution series in SV. Bottom row, left to right: *s*_fast_ and effective particle boundary amplitudes from the dilution series in SV, *s*_w_ and boundary fractions from a titration series in SV and steady-state fluorescence anisotropy competition titration.

**Figure 8:**
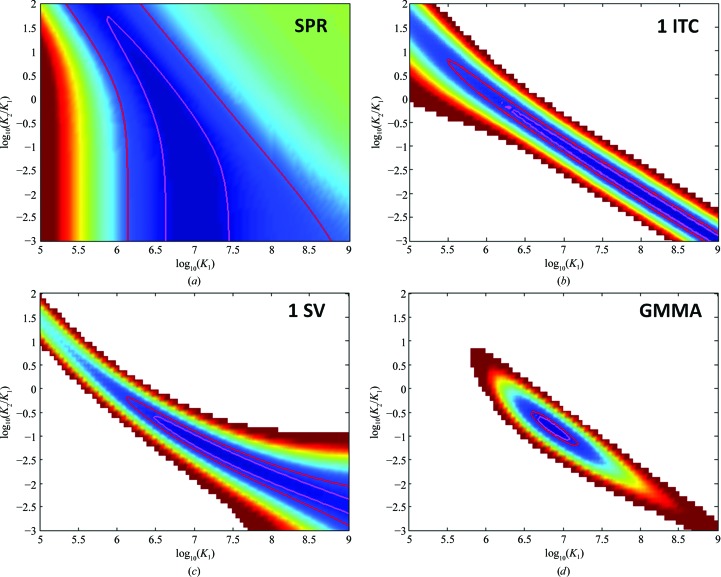
Parameter correlations between *K*_1_ and *K*_2_ for different experiments: (*a*) SPR alone (top left in Fig. 7 ▶), (*b*) a single ITC experiment (top second from right in Fig. 7 ▶), (*c*) a dilution series in SV (top right and first and second lower left in Fig. 7 ▶) and (*d*) GMMA jointly of the same data sets.

The premise of GMMA is that the combination of data sets from different techniques that exploit different observables for monitoring the same binding process can break the parameter correlation which exists in a single-method analysis, and thereby increases precision and opens up more complex multi-site and multi-component systems for study (Fig. 6 ▶). As an illustration of this principle, we applied GMMA to the two-site interaction of α-chymotrypsin binding soybean trypsin inhibitor (Fig. 7 ▶; Zhao & Schuck, 2012 ▶). Of the ITC, SPR, SV and fluorescence anisotropy methods applied, no single one was able to resolve the binding energies and enthalpies of the two sites, and the individual best-fit values were very different. Yet, in combination in GMMA they determined all of the binding parameters very well, as shown by the shapes of the error contours of the individual and GMMA analyses (Fig. 8 ▶). Remarkably, even though only ITC reports binding enthalpy changes and only SV reports sedimentation coefficients, these parameters of the 1:1 and 2:1 complexes were significantly better determined by GMMA than by the combination of ITC or SV alone or by single-method global analysis (Zhao & Schuck, 2012 ▶).

Modeling each technique involves global parameters {*p*_glob_}, which are dependent on the macromolecular interacting system, as well as local parameters {*p*_loc_}, which may be macromolecular properties that are only important in a subset of experiments (such as extinction coefficients or frictional coefficients) or technical ‘nuisance’ parameters such as instrumental baselines *etc*. *SEDPHAT* ‘projects’ an inter­action model into the different data spaces, determines the local root-mean-square deviation and χ^2^ of the fit and calculates from this a weighted overall χ^2^ as
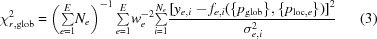
(where *f*_*e*,*i*_, *y*_*e*,*i*_ and σ_*e*,*i*_ are the model, data and standard deviation for data point *i* of experiment *e* out of a total of *N_e_* data points in a total of *E* experimental data sets, with a statistical weighting factor *w_e_* for each experiment). The object-oriented internal structure of *SEDPHAT* will automatically add and dynamically optimize the necessary local parameters for each data type when experimental data are added, without requiring any user intervention or data-specific or system-specific programming. Nonlinear regression then globally optimizes χ^2^_*r*,glob_ of the fit, adjusting the parameters (optionally with user-provided constraints or links between parameters) offering simplex, Marquardt–Levenberg and simulated-annealing algorithms.

### 3.2. Statistical analysis functions   

Standard statistical analysis functions are available in *SEDPHAT* to probe parameter errors and correlations in any fit, including cross-correlation parameters from the covariance matrix, contours of the error surface and its projections with F-statistics and Monte-Carlo analysis. For example, the reduction in parameter correlation between the *K*_d_ of the first and second binding site achieved through GMMA is visualized in Fig. 8 ▶ with two-dimensional projections of the error surface, which can be computed and displayed in *SEDPHAT*.

Specific for GMMA, we have introduced a statistical weight (*w_e_* in equation 3) to account for very dissimilar sized data sets and to ensure that all experiments can make statistical contributions to the global fit (Zhao & Schuck, 2012 ▶). Dependent on differences in the various techniques regarding the potential impact of systematic errors, this parameter can be adjusted by the user. A tool to scan the parameter errors as a function of the set of weights {*w_e_*} can alert as to whether any of the GMMA results is sensitive to this choice, and can report the overall largest confidence interval for all parameters (Zhao & Schuck, 2012 ▶). Furthermore, a statistical test based on cross-validation and F-statistics was implemented to flag data sets that do not seem to be mutually consistent, for example owing to possible differences from experimental imperfections or the influence of systematic errors. Finally, it is instructive to inspect which experiments carry significant information on each parameter. These functions are available in the Statistics menu of *SEDPHAT*, with the option to subgroup experimental data sets for the purpose of their statistical analysis.

### 3.3. Experimental design for GMMA using simulation tools   

It can be a nontrivial question what experiments should be performed for optimally probing binding equilibria when various methods are available. This is true, in particular, for multi-component and multi-site systems and in the context of GMMA. To facilitate experimental planning, we have implemented new simulation functions in *SEDPHAT* that can generate series of experimental data sets *in silico* that can be added to a GMMA. In conjunction with the statistical analysis functions of *SEDPHAT*, the user may probe which set of experiments and, for each technique, which experimental design would be most informative, given a certain hypothesis for the nature of the interactions and estimates of the likely binding constants. This may also reveal whether certain types of experiments would be worth conducting on the basis of their added information content, or whether it is possible to fully characterize a certain interacting system at all with the methods at hand and given the likely errors of data acquisition.

**Figure 9:**
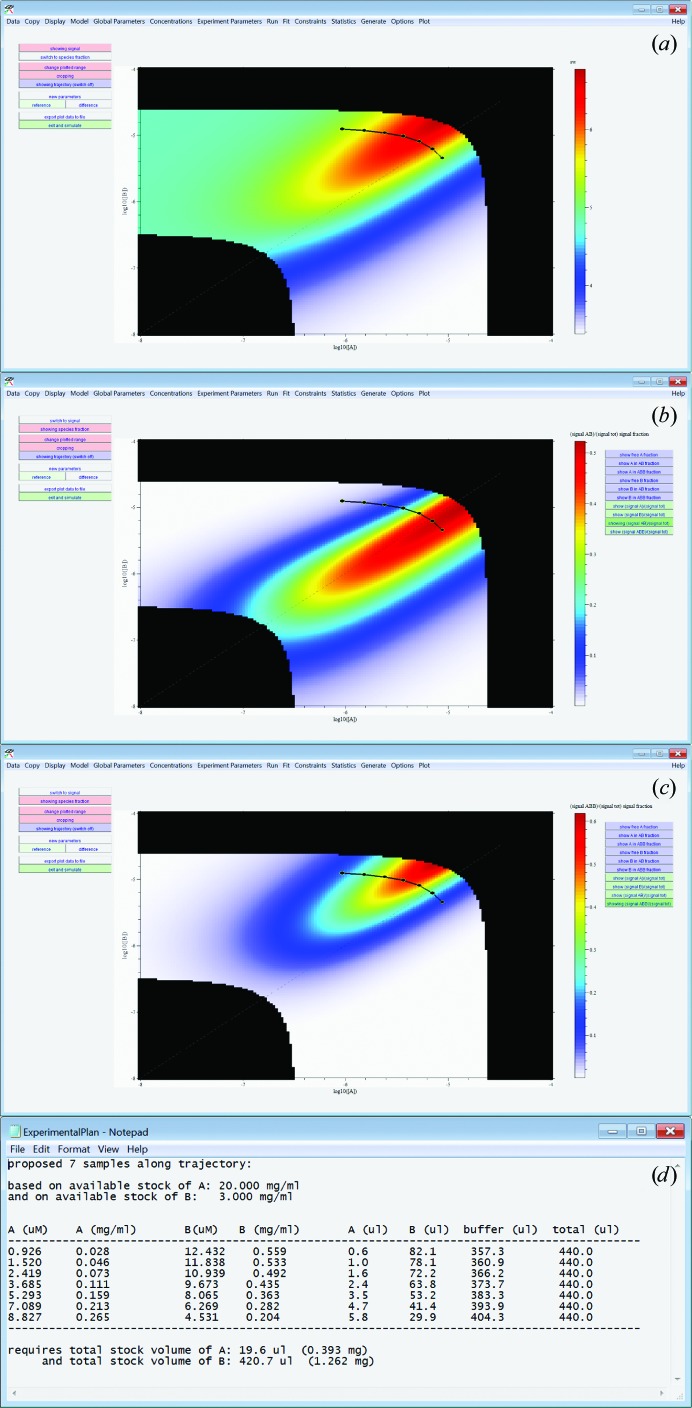
An example of detailed experimental planning for SV of a 2:1 binding system in *SEDPHAT*. (*a*) Parameter space of *s*_w_ isotherms for the given binding system as functions of macromolecular concentration for species A (abscissa) and B (ordinate), both in logarithmic units, and with the color temperature indicating the theoretical *s*_w_ values for given hypothesized binding constants, cropped to conditions where the total signal is between user-set parameters and experimentally feasible based on given stock concentrations. (*b*, *c*) Relative contribution of the AB and ABB complex, respectively, to the total signal. In all plots, a graphically user-generated trajectory (black line with points) is shown, which automatically generates the sample-preparation plan in (*d*).

To construct such a simulation in *SEDPHAT*, first the interaction model must be selected and global binding parameter estimates must be entered. The simulation functions can then be invoked to generate data sets of type SE, SV, SV isotherms, ITC, SPR, competition SPR, fluorescence anisotropy, general linear spectroscopy (such as steady-state fluorescence quenching) or any type of isotherms (for example chemical shift from NMR data and data of microscale thermophoresis). Likely values for local parameters specific to the particular technique must be provided (such as characteristic signal increments and/or ancillary experimental parameters). In order to help to create the most informative isotherms in the concentration space, the user is then brought to a two-dimensional map of either predicted total signal (for the given technique) or the fractional signal contributions of different complexes formed, or their fractional population as a function of total component concentrations (Fig. 9 ▶). Optionally, the field of view of this interaction map can be cropped to reflect feasible regions on the basis of maximal available stock concentrations and/or on the basis of minimal signals or maximal desired signals (such as OD limits in absorbance detection). Furthermore, the isotherm plots can be switched into a differential mode highlighting the regions of greatest sensitivity to changes in certain parameters. After visually discerning suitable isotherm trajectories from within any of these plots (which are presumably those that show characteristic changes along the trajectory, but this is to be assessed by statistical analysis and is likely to be dependent on the GMMA context), the user can draw a line with the mouse in the map and receive the desired number of log-equidistant mixture concentrations that probe this isotherm. For convenience, the user can obtain recipes to produce a certain volume of these mixtures from the previously given stock concentrations.

Constraining the concentration-dependent map of signal (or complex species) to feasible mixtures based on given component stock concentrations can highlight, for example, how high the stock concentrations should be to generate sufficient information and how much total volume would be required. With the caveat that the interaction parameters of the underlying simulations are hypothetical, these simulation tools can guide protein preparation and link it to experimental information content. Similarly, such simulations may reveal which signal-to-noise ratio would be required with a certain technique to be informative in the context of GMMA.

## 4. Concluding remarks   

*SEDPHAT* was designed as software that allows the seamless global analysis of a large number of experiments with different biophysical techniques without compromising the level of detail in the modeling of each technique. It has a graphical user interface and does not require any system-specific or data-specific programming, facilitating practical routine application in the laboratory. GMMA provides the opportunity to characterize more complicated systems of interacting proteins exhibiting multi-valency, multi-site interactions and/or cooperativity. We believe this is essential for fully understanding the quaternary structure and function of proteins in their signaling or regulatory pathways, adding complementary information to crystallographic structures.

## 5. Availability   

All of the tools and methods mentioned above are implemented in software that is available at no cost. *SEDPHAT* can be downloaded from https://sedfitsedphat.nibib.nih.gov/software/default.aspx and an online help system with some basic tuto­rials is available at http://www.analyticalultracentrifugation.com/sedphat/default.htm. *SEDPHAT* interfaces seamlessly with *NITPIC*, which reads raw thermogram data and supplies *SEDPHAT* ITC isotherm data files as an output. A Python version of *NITPIC* written by Dr Chad Brautigam can be downloaded from http://biophysics.swmed.edu/MBR/software.html. The advanced plotting program *GUSSI* can be found on the same webpage, also authored by Dr Chad Brautigam, which interfaces with *SEDPHAT* in many ways to easily achieve customizable publication-quality graphs of all data types. Finally, *EVILFIT* for the analysis of SPR kinetic surface-binding data is available from https://sedfitsedphat.nibib.nih.gov/software/default.aspx as a compiled *MATLAB* standalone executable with graphical user interface.

All of the software tools described in the present work, along with the principles and practice of the different biophysical techniques, are the subject of workshops that are held twice yearly, alternating at the National Institutes of Health in Bethesda, USA and at other national and international locations. Information about the workshops can be found at https://sedfitsedphat.nibib.nih.gov/workshop/default.aspx.
